# Design of the Physical exercise during Adjuvant Chemotherapy Effectiveness Study (PACES):A randomized controlled trial to evaluate effectiveness and cost-effectiveness of physical exercise in improving physical fitness and reducing fatigue

**DOI:** 10.1186/1471-2407-10-673

**Published:** 2010-12-07

**Authors:** Hanna van Waart, Martijn M Stuiver, Wim H van Harten, Gabe S Sonke, Neil K Aaronson

**Affiliations:** 1 The Netherlands Cancer Institute, Division of Psychosocial Research and Epidemiology, Amsterdam, The Netherlands; 2 The Netherlands Cancer Institute - Antoni van Leeuwenhoek Hospital, Department of Physical therapy, Amsterdam, The Netherlands; 3 The Netherlands Cancer Institute - Antoni van Leeuwenhoek Hospital, Department of Medical Oncology, Amsterdam, The Netherlands

## Abstract

**Background:**

Cancer chemotherapy is frequently associated with a decline in general physical condition, exercise tolerance, and muscle strength and with an increase in fatigue. While accumulating evidence suggests that physical activity and exercise interventions during chemotherapy treatment may contribute to maintaining cardiorespiratory fitness and strength, the results of studies conducted to date have not been consistent. Additional research is needed to determine the optimal intensity of exercise training programs in general and in particular the relative effectiveness of supervised, outpatient (hospital- or physical therapy practice-based) versus home-based programs.

**Methods:**

This multicenter, prospective, randomized trial will evaluate the effectiveness of a low to moderate intensity, home-based, self-management physical activity program, and a high intensity, structured, supervised exercise program, in maintaining or enhancing physical fitness (cardiorespiratory fitness and muscle strength), in minimizing fatigue and in enhancing the health-related quality of life (HRQoL). Patients receiving adjuvant chemotherapy for breast or colon cancer (n = 360) are being recruited from twelve hospitals in the Netherlands, and randomly allocated to one of the two treatment groups or to a 'usual care' control group. Performance-based and self-reported outcomes are assessed at baseline, at the end of chemotherapy and at six month follow-up.

**Discussion:**

This large, multicenter, randomized clinical trial will provide additional empirical evidence regarding the effectiveness of physical exercise during adjuvant chemotherapy in enhancing physical fitness, minimizing fatigue, and maintaining or enhancing patients' quality of life. If demonstrated to be effective, exercise intervention programs will be a welcome addition to the standard program of care offered to patients with cancer receiving chemotherapy.

**Trial registration:**

This study is registered at the Netherlands Trial Register (NTR 2159)

## Background

Treatment with chemotherapy is associated with multiple physical and psychosocial side effects, including reduced cardiorespiratory fitness and muscle strength and increased fatigue [1,2]. Fatigue is a common problem reported by patients undergoing chemotherapy, with prevalence rates ranging from 80% to 100% [3-5]. Among breast cancer survivors, the prevalence of chronic, severe fatigue has been reported to range from 24% to 40%. Chronic fatigue has a negative impact on activities of daily life, social reintegration and overall quality of life [3]. Fatigue and muscle wasting may be directly therapy-induced, but may also be attributed in part to sedentary habits and subsequent loss of physical fitness (cardiorespiratory fitness and muscle strength) [6].

There is accumulating evidence that exercise interventions during chemotherapy may contribute to preserving cardiorespiratory fitness [7-14] and muscle strength [13,15,16], decreasing fatigue [13,14,17-19], mood disturbances [13,14,17,18] and lean body mass [13], and enhancing self-reported functioning [18], overall HRQoL [13,14,18] and immune-function [17].

However, results of studies to date have not been entirely consistent [17,18,20], which may be due, in part, to methodological limitations. Most studies have employed small sample sizes (ranging from 6 to 60 patients in the intervention arm) and some have failed to include a control group. Furthermore, the interventions employed in these studies varied widely. Some studies have investigated low intensity, home-based exercise programs, while others report on intensive, structured and supervised training, with or without resistance training [14]. Most of the previous studies employed exercise programs that are less than optimal in terms of important aspects of exercise physiology [17]. It is hypothesized that an intervention that combines resistance training and aerobic exercise may be most effective [15].

Adherence to exercise interventions is a challenging task [20]. When shaping an exercise program to the needs of the individual patient, current fitness level, health beliefs and health behavior need to be taken into consideration, while still maintaining a core, standardized program that can be relatively easily implemented in a range of health care settings [20].

Effects of exercise interventions may be moderated by exercise history and health beliefs. It is likely that sociodemographic, medical variables, and patients' preferences also moderate the effects of exercise interventions during chemotherapy [21,22].

It should be noted that most trials are characterized by low participation rates (as low as 17% of potentially eligible patients), which may limit the generalizability of the results, particularly if those included in the trial tended to be more habitually active, more highly motivated and/or better educated than the target population as a whole [13,14].

Finally, to our knowledge, no study to date, has evaluated the cost-effectiveness of exercise interventions during chemotherapy.

In summary, while there is evidence supporting the beneficial effects of exercise programs during chemotherapy, the results across studies are not entirely consistent. Additional studies are needed to determine the optimal content, intensity, and form of exercise training programs. Specifically, there is need for research that investigates an exercise program that combines aerobic exercise and muscle strength training, as well as the relative effectiveness of supervised, outpatient versus unsupervised home-based programs. Such research should also consider the possible moderating effect of exercise history, current levels of fitness and physical activity, health attitudes, and motivation on physical exercise program effectiveness. The sample size and thus power of future trials should be sufficient to be able to demonstrate program effectiveness and cost-effectiveness based on both physiological performance measures, and fatigue.

In this article, we describe the design of a randomized, controlled, multicenter clinical trial comparing: (1) a low to moderate intensity, home-based, self-management physical activity program (Onco-Move), (2) a high intensity, structured, supervised exercise program (OnTrack) and (3) usual care control group, in patients undergoing adjuvant chemotherapy for breast or colon cancer.

We hypothesize that patients who undergo the Onco-Move or the OnTrack program will achieve better physical fitness levels, as assessed by objective performance tests, will report less fatigue, less mood disturbance, higher levels of physical activity and functioning in daily life, and better HRQoL than patients in the usual care control group. Furthermore, we hypothesize that patients in the OnTrack program will achieve more muscle strength and achieve greater gains in cardiorespiratory fitness than patients who follow the Onco-Move program. No differences are expected between the two programs in self-reported outcomes during treatment. However, it is hypothesized that, at the six month follow-up, patients who participated in the OnTrack program will report less fatigue, and higher levels of physical activity and functioning than those who participated in the Onco-Move program or usual care control group. Finally, we hypothesize that the OnTrack and Onco-Move programs will lead to a reduction in health care costs, patient and family costs, and costs of production losses, resulting in an cost-effective intervention.

If demonstrated to be effective, the availability of such physical activity and exercise intervention programs will be a welcome addition to the standard program of care offered to patients with cancer undergoing chemotherapy.

## Methods

**Figure 1:**
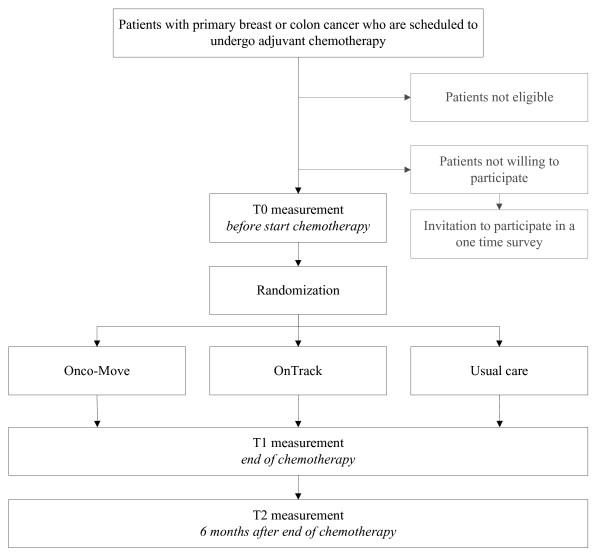
**Overview of study procedures**.

The present trial is one of four randomized controlled trials included in the Alpe d'HuZes Cancer Rehabilitation (A-CaRe) program. All studies within the A-CaRe program will evaluate the effectiveness and the cost-effectiveness of exercise-based rehabilitation interventions in different cancer patient and survivor groups. In the PACES study, patients are being randomized to one of three study arms. They will participate either in the Onco-Move or the OnTrack program, or will undergo usual care. The design of the trial and the anticipated flow of the participants are displayed graphically in Figure 1. The trial has been approved by the institutional review board of the Netherlands Cancer Institute (under number PTC 09.2711), as well as by the review boards of all hospitals from which patients are being recruited. This protocol follows the CONSORT guidelines [23]. Patient recruitment and data collection for this trial started in April, 2010.

### Study sample

The study sample will be composed of patients with histologically confirmed primary breast or colon cancer who are scheduled to undergo adjuvant chemotherapy. There is no upper age limit for study participation.

Potentially eligible patients are screened for the presence of comorbid conditions that would contraindicate participation in a physical activity/exercise program. This includes patients with serious orthopedic conditions that would hamper functional recovery, and patients with serious cardiovascular or cardiopulmonary conditions (or risks) who would not be able to train at the intensity level required. Patients suffering from malnutrition as evidenced by a BMI < 18 kg/m^2^, unintended weight loss of more than 5% per month, or more than 10% unintended weight loss during the previous six month period are considered poor candidates for physical condition training and thus are not eligible for participation. Patients judged to have serious psychiatric or cognitive problems that would preclude them from program participation are excluded from the study. For assessment purposes, study participants need to have basic fluency in the Dutch language. Finally, patients participating in concurrent studies or rehabilitation programs containing elements of physical activity or exercise are also ineligible for the study.

### Recruitment and randomization

In total 360 patients are being recruited from twelve community or university hospitals in the wider Amsterdam region of the Netherlands. All potentially eligible patients are identified by their medical oncologist or nurse practitioner, and receive written information about the trial. The patients are contacted by telephone to provide additional information about the trial and to confirm their eligibility. If the patient chooses to participate in the trial, (s)he is invited for an intake session where written informed consent is obtained and baseline assessments are made. Subsequently, patients are assigned to one of the three study groups by means of the minimization method [24], which is balanced on age, primary diagnosis, treating hospital and the use of trastuzumab.

Patients who decline to participate in the trial are asked to complete a one-time questionnaire assessing basic sociodemographic, attitudinal, and behavioral data, and to determine the reason(s) for non-participation.

### Interventions

Patients are randomized to one of three study groups: Onco-Move, OnTrack, or usual care. The Onco-Move program aims at preserving cardiorespiratory fitness, as advocated by Mock [25]. The OnTrack program combines exercise for both cardiorespiratory fitness and muscle strength, as advocated by Courneya [15]. Both the Onco-Move and OnTrack program start in the week of the first cycle of chemotherapy and continue until three weeks after the last cycle of chemotherapy. At the end of chemotherapy all patients receive a leaflet providing encouragement and information on ways to be active.

#### Onco-Move

Onco-Move is a relatively low intensity, home-based, individualized, self-managed physical activity program developed and tested in a pilot study by the Comprehensive Cancer Centre Amsterdam (IKA). Based on the "Every Step Counts" program of Mock [25,26], it uses self-management principles, aiming to maintain general physical fitness and preventing fatigue.

Trained nurses encourage patients to pursue an active lifestyle, 30 minutes per day, throughout the chemotherapy treatment, starting at the first chemotherapy session. Activities depend on patient preference, which may include walking, cycling, fitness training or swimming. Training with weights is not encouraged. At the first chemotherapy session, patients receive both verbal and written information about physical activity training, and specific advice about the desired intensity of activity based on the Borg Scale of perceived exertion (recommended level 12-14) [27]. The written information is based on the transtheoretical model of behavior [28,29], called "active living" which identifies different stages of activity (pre-contemplation, contemplation, action, and maintenance).

The information patients receive about physical activity is tailored to their activity stage. For example, patients in the contemplation stage receive more information on why and how to become active, while patients in the maintenance stage receive information on how to stay active [28,29]. Two weeks after the start of the program the nurse contacts the patient by telephone to inquire about how the program is going and if there is any additional information or advice needed. At each subsequent chemotherapy cycle, the nurse discusses program progress with the patient. Patients are asked to keep daily activity diaries, both to help them to monitor their own progress, and as an aid for the nurse to facilitate optimal use of the program.

#### OnTrack

The OnTrack is a relatively high intensity exercise program which is supervised by a physical therapist in an outpatient or general physical therapy practice setting. The pilot study showed that OnTrack was feasible in patients undergoing chemotherapy [30]. We are making use of the Onconet network of physical therapists in the region of North Holland who received special training in the OnTrack program. This facilitates patients to undergo the OnTrack program as close to home as possible.

In this program, patients are encouraged to be physically active for at least 30 minutes per day at Borg level 12-14 [27]. Like the Onco-Move program, patients are asked to keep a daily activity dairy. The physical therapists use the "active living" method to encourage daily physical activity. The physical therapist reviews the daily activity diary with the patient every three weeks.

Additionally, patients attend supervised exercise sessions two times per week. These sessions comprise exercises for warming up followed by exercises to maintain or increase muscle strength and exercises to maintain or increase aerobic capacity. Muscle strength exercises are performed for 20 minutes per session, starting with two series of 12 repetitions at 70% of the one repetition maximum (1RM) per exercise and increasing gradually to two series of eight repetitions at 80%1RM. Exercising in sets of two series is considered a sufficient, yet time efficient means of enhancing muscle strength, ensuring that all exercises can be performed during a session [31]. The resistance program consists of at least six exercises targeting the large muscle groups as follows: 1) vertical row (longissimus, biceps brachii, rhomboideus); 2) leg press (quadriceps, glutei, gastrocnemius); 3) bench press (pectoralis major, triceps); 4) pull over (pectoralis, triceps brachii, deltoideus, trapezius); 5) abdominal crunch (rectus abdominis); and 6) lunge (quadriceps, glutei, hamstrings). Additional exercises can be added according to patients' preferences.

For patients with breast cancer who have had axillary lymph node dissection, a modified strength training program is used for the upper extremities, consisting of two series of 15 repetitions for each exercise with the lightest possible weight, which is increased with the smallest possible step when the two series can be completed without symptoms of lymph edema [32].

Aerobic exercises are performed for 30 minutes per session (with a minimal duration of ten minutes per exercise), with an intensity of 50% to 80% of the maximal workload (Wmax) as estimated by the Steep Ramp Test [33]. The intensity of the aerobic exercises are increased if a patient scores a 12 or lower on the Borg scale of perceived exertion, while the intensity is decreased at a score of 16 or higher [27]. The heart rate should be within a heart rate zone of 60% to 90% of the maximal heart rate, which is conventionally estimated as 220 minus age.

#### Usual care

Usual care will vary according to hospital guidelines and doctors' and patients' preferences. Although usual care can not be standardized, it will not involve routine, systematic exercises.

### Study outcomes

All study outcomes, with the exception of return to work, compliance and satisfaction with the interventions, are assessed prior to randomization (T0), at the completion of chemotherapy (T1), and at 6 month follow-up (T2). Return to work is assessed at T1 and T2, while compliance and satisfaction are assessed at T1 only, and only in the two intervention groups.

To facilitate comparison of results across the A-CaRe studies, the outcome measures used in these four clinical trials have been harmonized to as great an extent as possible.

### Primary outcome measures

#### Cardiorespiratory fitness

Cardiorespiratory fitness is being assessed with the steep ramp test (an incremental bicycle ergometer test) and a cycle endurance test. Both tests are completed on a calibrated, electronically-braked cycle ergometer (Corival, Lode, Groningen, The Netherlands). For the steep ramp test, the patient is instructed to cycle at a speed between 70 and 80 revolutions per minute (RPM), starting with a 3 minutes warming-up at 10 Watts. The test starts at 25 Watts, after which the load is increased by 25 Watts every 10 seconds. The test ends if cycling speed falls below 60 RPM. Maximal workload (Wmax), time cycled at that load, and heart rate at the end of the test are recorded. The steep ramp test has been shown to be a reliable (ICC = 0.996) and valid (correlation with peak oxygen uptake (peakVO_2_) of 0.85) means of estimating maximal workload in patients with cancer. PeakVO_2 _can be estimated from the steep ramp test using a linear regression equation [33].

Exercise endurance is measured after a period of rest following the steep ramp test. The cycle endurance test is done at a workload based on 70% of the Wmax reached during the steep ramp test. After a one-minute warming-up on the same ergometer that was used for the steep ramp test, the load is increased to the predetermined workload. The patient continues cycling at this constant submaximal workload until the cycling speed falls below 60 RPM, with a maximum time of 30 minutes. The endurance time and heart rate at the end of the test are recorded. The workload of the endurance test used at follow up measurements is the same as that of the baseline endurance test.

#### Muscle strength

Upper extremity muscle strength is measured with the JAMAR^® ^grip strength dynamometer [34] and the microFET^® ^hand held dynamometer (HHD) for the elbow flexion, using a standardized measurement protocol [35]. Lower extremity muscle strength is tested with the microFET^® ^HHD for the extension of the knee, again using a standardized measurement protocol [36], and with the 30 s chair stands test. During the 30 s chair stands test patients are asked to stand up from a chair with their arms folded across the chest, then to sit down again. The action is repeated at their fastest pace over a 30 second period. The final test score is the number of times that the patient rises to a full stand from the seated position with arms folded within 30 s. The 30 s chair stands test has been shown to be a valid and reliable measure of proximal lower limb strength in older adults [37].

#### Fatigue

Fatigue symptoms are assessed with the Multidimensional Fatigue Inventory (MFI) [38]. The MFI is composed of 20 items, organized into five scales: general fatigue, physical fatigue, reduced activity, reduced motivation, and mental fatigue. Questions are posed about the past few days, and responses are recorded on a five-point scale.

In addition to the MFI patients are asked to complete the Fatigue Quality List (FQL) [39], assessing patients' perception and appraisal of experienced fatigue. The FQL consists of 25 adjectives describing the fatigue experience, organized into 4 subscales: frustrating, exhausting, pleasant, and frightening.

### Secondary study outcomes

Secondary outcomes are mood disturbance, quality of sleep, health-related quality of life, functioning in daily life, measured physical activity level, self-reported physical activity level, anthropometric measures, return to work, chemotherapy completion rates, compliance, satisfaction with the intervention, adverse effects and costs from a societal perspective. A complete overview of assessments and instruments are presented in Table 1. A small selection of these measures is described in detail.

**Table 1:** Outcome measures

Assessment	Measurement instrument
**Primary Outcome measures**	

Cardiorespiratory fitness	Steep Ramp Test [33] Endurance Test

Upper Muscle Strength	JAMAR^® ^grip strength dynamometer [34] microFET^® ^HHD elbow flexion [35]

Lower Muscle Strength	microFET^® ^HHD extension knee [36] 30 s chair stands test [37]

Fatigue	MFI [38] FQL [39]

**Secondary Outcome measures**	

Mood disturbance	Hospital Anxiety and Depression Scale (HADS) [52]

Quality of sleep	Pittsburgh Sleep Quality Index (PSQI) [53]

Health-related quality of life	EORTC QLQ-C30 [32]

Functioning in daily life	Impact on Participation and Autonomy (IPA) [54]

Measured physical activity level	Actigraph accelerometer

Self-reported physical activity level	Physical Activity Scale for the Elderly (PASE) [55]

Anthropometric measures	Skinfold measurements (Harpenden) Waist and hip circumferences [56]

Return to work	Return to work questionnaire

Chemotherapy completion rates	Medical records

Compliance	Number of sessions attended and activity diary

Satisfaction with the intervention	Satisfaction questionnaire

Adverse effects	Medical records

Costs from a societal perspective	EuroQol EQ5D [42] and monthly cost diaries

#### Measured physical activity level

The level of physical activity will be objectively measured with the Actigraph (Actigraph, Fort Walton Beach Florida, USA), a small accelerometer which can measure accelerations from 0.05 to 2.00 G [40]. These accelerations are scored in counts per minute that provide information about how long and how intensive a patient has been physically active. The epoch will be set at 5 seconds. Patients wear the accelerometer on the right hip for 5 days including at least one weekend day. The Actigraph will not give any form of feedback to the participant.

#### Chemotherapy completion rates

Chemotherapy completion rate will be assessed as the average relative dose-intensity for the originally planned regimen based on standard formulas [41]. The data will be obtained via medical records.

#### Costs from a societal perspective

For cost-utility purposes, the EQ-5D, a brief HRQoL measurement, is included in the questionnaire package [42]. Health care costs, patient and family costs, and production losses will be assessed, and relevant data will be collected thought retrospective cost diaries measured on a monthly basis during the entire study period.

### Sociodemographic and clinical data

At baseline, sociodemographic data (e.g., age, gender, education, marital status, living and work situation), and lifestyle variables (e.g., smoking history), are being obtained via questionnaire.

Clinical information, including (date of) diagnosis, tumor characteristics, treatment (e.g., type of surgery, chemotherapy regimen), hemoglobin levels and medication use will be abstracted from the medical records. During the follow-up period, data on disease status (progression/recurrence) and any additional treatment (e.g., endocrine therapy, trastuzumab) is obtained via medical records and self-report.

### Moderating variables

A series of questions is posed to assess potential moderating variables, and variables that may be predictive of compliance with the physical exercise and activity programs. These include items assessing attitudes towards and beliefs about physical activity, social influence from peers, barriers to and perceived benefits of physical activity, self-efficay towards physical activity and stage of change [43-46]. Patients' preferences for type of exercise intervention is also being assessed [21].

### Non-participant analysis

In previous studies of exercise programs among patients with cancer, the generalizability of the results was limited due to a relatively low participation or uptake rate [13,14]. We expect that, in the current trial, a substantial percentage of eligible patients will decline the invitation to participate in the trial. We hypothesize that patients who choose not to participate have been less physical active and have led a more sedentary lifestyle prior to becoming ill than those who participate. We also expect that they have less favorable attitudes towards physical exercise, in general, and during chemotherapy treatment, in particular. Sociodemographic characteristics, fatigue and attitudes and behaviors with regard to physical exercise of the non-participants will be compared with those of the trial participants, using appropriate statistics (e.g. students t-test, chi square, etc.).

### Power calculation

The primary study outcomes include two performance measures (cardiorespiratory fitness and muscle strength) and self-reported fatigue. It is expected that patients in the usual care condition will experience a 5%-10% decline in physical fitness during their chemotherapy treatment, that this will improve gradually during the post-treatment period, but will not necessarily return to pretreatment levels. It is hypothesized that the OnTrack program will yield at least a 20% improvement in general physical fitness. Based on the results of Courneya et al., a pretreatment mean for the 1-repetition maximum for leg extension is estimated to be 25 kg, with a standard deviation of 12 kg [15]. We expect that the Onco-Move program will help patients to maintain their pretreatment levels of cardiorespiratory fitness, but not necessarily improve, and that it will have little or no effect on muscle strength.

Based on the above figures, 100 patients per group are needed to detect a 0.40 standard deviation difference (Cohen's effect size [47]) in performance-based outcomes between the OnTrack group and the usual care group at the post-chemotherapy assessment, with power set at 0.80 and alpha at 0.05 (two-sided test).

We will recruit 360 patients into the trial to allow for an attrition of approximately 20% (i.e., patients who discontinue participation in the trial entirely, including failure to complete follow-up assessments). These numbers will be sufficient to detect a one-half standard deviation unit difference in self-reported fatigue. This magnitude of difference is generally considered to be indicative of clinically meaningful differences in patients' self-reported symptom experience [47,48].

### Statistical analyses

All primary statistical analyses will be conducted on an intention-to-treat basis. Between group differences over time in performance indicators of general physical condition and muscle strength will be evaluated using multilevel regression analysis for % change from baseline, and two factor (group × time) multivariate analysis of variance with repeated measures on the time factor for comparison of mean scores. Scores on the self-report measures of fatigue, mood state and HRQoL will be calculated according to published scoring algorithms. Between-group differences over time in mean scores will be tested using a two factor (group × time) multivariate analysis of variance with repeated measures on the time factor. Effect sizes will be calculated using standard statistical procedures.

Supplementary analyses will be carried out in which data relating to compliance with the program elements are taken into account. Specifically, we will determine whether the level of compliance (based on attendance records and self-report data) is associated significantly with the changes over time in physical condition, muscle strength, fatigue, mood, and HRQoL. Similarly, we will investigate whether program effectiveness varies significantly as a function of patients' background characteristics, and particularly those variables assessing life style, health behavior and health attitudes.

### Cost-effectiveness analysis

This study also includes both incremental cost-effectiveness and cost-utility analyses. The cost-effectiveness ratio is calculated by dividing the difference between the mean total costs of the exercise and control groups by the difference in mean primary clinical effects of the groups [49]. In this analysis both direct and indirect costs will be taken into account. The incremental cost-utility ratio expresses the additional costs of the intervention per quality-adjusted life year (QALY) gained, compared to the usual care control group.

## Discussion

Compromised physical fitness and increasing fatigue are common side effects of cancer chemotherapy. Exercise during chemotherapy is a promising strategy for intervening at any earlier stage, to minimize or even prevent side effects both in the short- and long-term. Previous studies investigating the value of physical exercise during treatment have yielded inconsistent results, and many studies exhibited a range of methodological limitations.

In the current trial, we are evaluating the effectiveness and cost-effectiveness of a low intensity, home-based physical activity program, and a structured, supervised, moderate intensity exercise program in maintaining or enhancing physical fitness (cardiorespiratory fitness and muscle strength), in minimizing fatigue, and in enhancing HRQoL of patients undergoing adjuvant chemotherapy for breast cancer or colon cancer.

Our trial has several strengths, including: (1) the randomized trial design; (2) the multicenter nature of the trial; (3) the large sample size; (4) the relatively long-term follow-up; (5) the head-to-head comparison of two interventions varying in nature and intensity; (6) inclusion of both performance based and self-reported outcomes; (7) the use of intention-to-treat analysis; (8) the inclusion of a cost-effectiveness and cost-utility evaluation; and (9) the detailed evaluation of the background, attitudes and behavior of patients who decline to participate in the trial.

Several limitations of the trial should also be noted. First, although direct peakVO_2 _measurements are considered the gold standard for assessing cardiorespiratory fitness, these are not feasible in our trial because of the number and geographical spread of the training locations, and the (travel) time and costs involved with centralized measurements. However, it can be argued that an improvement in cycle endurance time may be more clinically relevant than an improvement in peakVO_2 _[50,51]. Second, although the usual care control group will not be provided with any materials or programs elements relating to physical exercise, clinicians and ancillary health care providers are increasingly recognizing the potential value of physical activity both during and following cancer treatment. Thus we cannot rule out the possibility that some patients in the control group will be encouraged to increase their level of physical activity, either by their caregivers or via the media. Nevertheless, we do not anticipate that this will take place in a structured or systematic way, and thus the planned comparisons (between the two intervention groups and the control group) will still be valid.

In summary, given the increasing number of patients with cancer, and improving survival rates, it is important to ensure that patients' physical and psychosocial health is maintained or even enhanced to as great an extent as possible both during active treatment and once treatment has been completed. Encouraging and facilitating physical activity during treatment may enhance health outcomes in both the short- and long-term.

## List of abbreviations

PACES: Physical exercise during Adjuvant Chemotherapy Effectiveness Study; A-CaRe: Alpe d'HuZes Cancer Rehabilitation; HRQoL: Health-related quality of life; RPM: revolutions per minute; Wmax: Maximal workload; peakVO_2_: peak oxygen uptake; HHD: hand held dynamometer; QALY: quality-adjusted life year

## Competing interests

The authors declare that they have no competing interests.

## Authors' contributions

NA, WvH, MS and GS are the principal investigators of this trial. HvW is the PhD student of this trial, and generated the first draft of this manuscript based on the original study protocol. All authors approved the final version of the manuscript.

## Pre-publication history

The pre-publication history for this paper can be accessed here:

http://www.biomedcentral.com/1471-2407/10/673/prepub
